# Eosinophilic Gastroenteritis With Malabsorption, Acute Intestinal Obstruction, Ascites and Pleural Effusion: A Case Report and Review of Literature

**DOI:** 10.4021/gr586w

**Published:** 2014-01-15

**Authors:** Aloisio Antonio Gomes de Matos Brasil, Luiza Neves Pinheiro Bezerra, Estela Lucena Alcantara Bruno, Danyelle Rolim Carvalho, Paulo Levi Pereira de Oliveira, Roana Lacerda Tavares Leite

**Affiliations:** a Department of Internal Medicine of the Medical College of Cariri, University of Ceara, Barbalha CE, Brazil

**Keywords:** Eosinophilic gastroenteritis, Acute obstructive abdomen, Ascites, Pleural effusion, Immunoglobulin E, D-xylose absorption test

## Abstract

We report a case of a 49-year-old male patient with abdominal distension and diffuse stomach cramps associated with peripheral eosinophilia. Treatment for eosinophilic parasitosis was not effective. After a few weeks, the patient developed acute obstructive abdomen with ascites, which was atypically improved with the use of antispasmodics and analgesics. Upper digestive endoscopy, colonoscopy and histopathologic examination of the gastric and intestinal mucosa did not show any significant changes. Video laparoscopic biopsy of the mesenteric lymph node and peritoneum revealed a nonspecific chronic inflammatory process with intense diffuse tissue eosinophilia. Complementary tests revealed right-sided pleural effusion and increased serum immunoglobulin E levels, with altered D-xylose absorption test results. The patient was treated with a hypoallergenic diet and an oral corticosteroid; the symptoms resolved and the laboratory test results improved. Eosinophilic gastroenteritis is a rare inflammatory disease characterized by eosinophilic infiltration in the wall of the gastrointestinal tract. The clinical presentation varies according to the affected site and the depth and extent of digestive tract involvement. This case report, which presents the rare simultaneous involvement of the mucosal, muscular and serosal layers, aims to describe and discuss the clinical and therapeutic aspects of eosinophilic gastroenteritis as well as its progression.

## Introduction

Eosinophilic gastroenteritis is a rare inflammatory disease characterized by eosinophilic infiltration in the gastrointestinal tract affecting all age and ethnic groups [1]. In adults, the disease usually manifests between the third and fifth decades of life [2]. It can affect any part of the digestive tract from the esophagus to the rectum. The most commonly affected segments are the stomach, in particular the antrum, and the small intestine [3]. The clinical presentation depends on the site and depth of eosinophilic infiltration [2]. Kaijser described the first case of this disease in 1937, and since then, approximately 300 cases have been reported in the literature [3-5].

## Case Report

A 49-year-old male patient belonging to a mixed race presented with a complaint of postprandial bloating, abdominal distension and diffuse stomach cramps of moderate intensity since 2 weeks ago. He reported a history of allergic rhinitis, lactose intolerance, social drinking and smoking, the lattermost being discontinued since 10 years ago. He was not consuming any medications. The patient was treated for intestinal parasitosis (albendazol, 400 mg/day for 3 days) on an outpatient basis; however, this treatment proved ineffective. Complementary tests revealed leukocytosis (13,500/mm^3^) with normal neutrophils, no left shift, no relative and absolute eosinophilia (16% and 2,160/mm^3^). Erythrocyte and platelet counts, renal function, fasting glucose levels and lipid profile were normal. Liver enzymes were elevated (TGO, 95 U/L; TGP, 92 U/L; GAMA-GT, 145 U/L; alkaline phosphatase, 129 U/L). Three parasitology samples, coprology and stool culture yielded negative results.

A month after the onset of symptoms and several emergency care visits, the patient’s condition worsened, with the onset of acute intestinal obstruction. He presented with increased abdominal distension associated with intense pain, nausea, vomiting, and blocked stool and gas flow, which atypically was improved with analgesic, antispasmodic and antiemetic drugs. No weight loss was observed. Physical examination revealed a poor general condition with mild dyspnea, flushing and dehydration. He was afebrile, anicteric and acyanotic. His abdomen was swollen, tense, and diffusely painful on superficial and deep palpation, with a positive Blumberg sign, active bowel sounds and no visceromegaly. Lung auscultation revealed decreased vesicular murmurs at the right lung base, and cardiovascular examination showed no changes. The patient’s vital signs were normal.

The patient was hospitalized for investigations, and complementary tests were performed. Laboratory tests confirmed relative and absolute eosinophilia (26% and 9,400/mm^3^), elevated inflammatory markers (ESR, 70 mm/h; RCP, 8.9 mg/dL), and decreased total protein (5.9 g/dL) and albumin (3.3 g/dL) levels. The serum potassium level was slightly decreased (3.46 mmol/L), and serum sodium level was 135.0 mmol/L. The test for carcinoembryonic antigen (CEA) was negative (0.2 ng/mL). A urine test revealed slightly cloudy urine with traces of proteins and ketone bodies (++). A urine culture tested negative.

**Figure 1:**
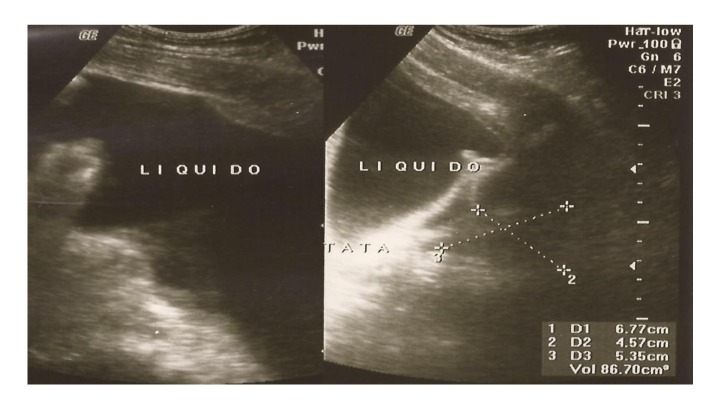
Abdominal ultrasound with a large volume of ascitic fluid in the peritoneal cavity.

Abdominal ultrasound revealed grade III hepatic steatosis and a large amount of intraperitoneal free fluid (Fig. 1).

Computed tomography and magnetic resonance imaging of the entire abdomen revealed diffuse thickening of the mesenterium with vessel ectasia, which was compatible with mesenteric panniculitis and was possibly associated with sclerosing mesenteritis, large-volume ascites, moderate right-sided pleural effusion (800 mL) and bilateral simple renal cysts measuring approximately 1 cm (BOSNIAK 1).

**Figure 2:**
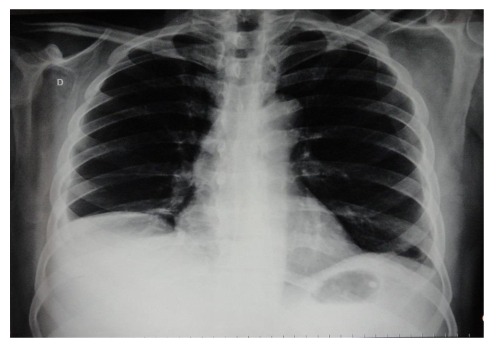
A chest X-ray showing moderate, right-sided pleural effusion.

Chest radiography revealed moderate right-sided pleural effusion without parenchymal changes (Fig. 2).

Upper digestive endoscopy revealed a normal esophagus and duodenum, and mild enanthematous pangastritis. A urease test was negative. Video colonoscopy did not show any abnormalities. Gastric and intestinal biopsies revealed mild, nonspecific, chronic inflammation with normal eosinophil numbers in the mucosa.

Paracentesis revealed an albumin level of 1.150 g/dL, an increased glucose level (108.0 mg/dL), a lactic dehydrogenase level of 446.0 U/L, an adenosine deaminase level of 20.3 U/L and a pH of 8.0. Culture yielded negative results.

A diagnostic, video-assisted laparoscopy with biopsy was performed. Histopathologic examination of the peritoneum and mesenteric lymph nodes revealed a nonspecific, chronic inflammatory process with intense, diffuse, eosinophilic infiltration (48/field and 35/field, respectively), few lymphocytes and macrophages, extensive fibrosis of the subperitoneal connective-adipose tissue, and fibrin deposition on the surface. There were no signs of malignancy.

Eosinophilic gastroenteritis was suspected, and total immunoglobulin E levels were measured and found to be elevated (543 KU/L). The D-xylose test was also performed; the results confirmed signs of malabsorption (17.0 mg/dL).

A diet restricted in soy, wheat, corn, milk and its derivatives, eggs and seafood was prescribed and prednisone was initiated at 30 mg/VO/day. The patient was discharged after the symptoms were relieved, with a prescription of oral corticosteroid therapy that was to be tapered over a month.

The patient has been followed on an outpatient basis for a year and has been asymptomatic till date.

## Discussion

Eosinophilic gastroenteritis predominantly affects men, and on average, 70% patients have a personal or family history of atopy [2, 6, 7]. Food allergies or immune anomalies have been determined to be its main etiologies and are reported in 25%-75% patients [4, 6]. Moreover, some studies reported eosinophilic gastroenteritis caused by medications such as gold salts, enalapril, azathioprine, co-trimoxazole, gemfibrozil and carbamazepine [3, 4, 8-10].

The diagnostic criteria include the presence of gastrointestinal symptoms and peripheral eosinophilia in up to 80% patients, with no parasitosis or extraintestinal diseases [2, 3, 11]. An increase in immunoglobulin E levels may also be associated [4, 11, 12].

The present report presents epidemiologic, clinical and laboratory aspects that are in line with those reported in the literature. Moreover, a personal history of atopy and elevated immunoglobulin E levels confirm the allergic nature of the process [4, 11, 12]. Although some studies indicate medications as the cause of the disease, there was no such history in our patients.

According to histopathologic findings, the eosinophilic infiltrate in the gastrointestinal tract is pathognomomic. The precise number of eosinophils, which is used as a criterion to define the disease, remains controversial [1, 13]. In addition, false-negative results from biopsies of the gastric and intestinal mucosa may occur because the eosinophils may either be sparse or clustered in the deeper layers of the walls with affected mucosa.

There is a loss of integrity in the intestinal barrier in predisposed individuals. This allows antigens to cross the mucosa, thus inducing degranulation of mast cells, which release chemotactic factors that recruit eosinophils. The gastrointestinal tract is thus susceptible to direct damage by eosinophils via the release of toxic proteins (major basic protein and eosinophil peroxidase) as well as indirect damage via leukotrienes, release of histamine and cytokines (IL-2, IL-3, IL-4, IL-5), tumor necrosis factor alpha (TNF-a), granulocyte-macrophage colony-stimulating factor (GM-CSF) and transforming growth factor beta (TGF-b) [4, 8, 11, 14].

Eosinophilic gastroenteritis has been classified into mucosal, muscular and serosal types according to the predominantly affected layer as per histopathologic examination; these present specific differences in clinical manifestations [3, 4, 14, 15]. The mucosal type of the disease is the most common form (25%-100% patients). Symptoms include diarrhea, abdominal pain, nausea, vomiting, weight loss and gastrointestinal bleeding. In the more advanced stages, the malabsorption syndrome may be observed, which can be confirmed by changes in the D-xylose test. Endoscopic examination may show thickening of the mucosal folds, polyps, luminal narrowing and ulcerations. The muscular type of the disease (13%-70% patients) is characterized by typical symptoms of intestinal obstruction caused by the thickening and rigidity of the muscular layer and may be accompanied by dysmotility symptoms such as nausea, vomiting, abdominal distension and pain. The serosal type of the disease (12%-40% patients) is related to eosinophilic ascites, adherences, omental thickening, eosinophilic lymphadenopathy and increased peripheral eosinophilia. Pleural effusion or increased eosinophil counts may be revealed by biopsy of pleural specimens. Simultaneous affection of the three layers is not common [4].

The patient exhibited symptoms that were typical of concomitant mucosal, muscular and serosal involvement, with an emphasis on malabsorption associated with acute obstructive abdomen, large-volume ascites, pleural effusion and significant peripheral eosinophilia. The peritoneum and lymph node biopsies showing intense eosinophilic infiltration explain the presence of ascites and pleural effusion as detected by imaging, even in the absence of eosinophils in ascitic and pleural fluids and/or pleural specimens obtained by biopsy.

The pharmacologic treatment of choice is the use of corticosteroids: prednisone, 20 - 40 mg/VO/day for 1 - 2 weeks, followed by withdrawal or maintenance with a dosage of 5 - 10 mg/day. Prednisolone at 40 - 60 mg/VO/day or hydrocortisone at 200 - 400 mg/IV/day can also be prescribed. The use of antiallergic drugs such as sodium chromoglycate (200 mg/6 - 6 h), ketotifen (2 - 4 mg/day), sodium montelukast (20 - 30 mg/day) and suplatast tosilate (300 mg/day), with or without corticosteroids, has been reported to be relatively successful [11, 16]. Other drugs are also reported for use in corticosteroid-dependent or corticosteroid-resistant cases, with some showing discrepant results, such as azathioprine, cyclophosphamide, 6-mercaptopurine, cyclosporine A, hydroxyurea, omalizumab and mepolizumab [3, 11, 17, 18]. Finally, diet management may lead to disease remission in rare cases where the mucosa is predominantly involved and a specific food intolerance or allergy has been identified [11].

It was very important for the patient to associate diet management with the use of prednisone, which facilitated relief from symptoms and normalization of the number of eosinophils in our patient. There were no relapses after corticosteroid withdrawal.

Complications such as obstruction, perforation, suspicion of cancer and refractoriness to drugs are an indication for surgical intervention [1, 19]. The prognosis is benign in most cases. There are no reports of long-term sequelae, a higher probability of developing cancer, or decreased life expectancy [5, 11].

We conclude that limited knowledge about the characteristics of eosinophilic gastroenteritis and the paucity of evidence may lead to misdiagnosis or inadequate therapy such as emergency surgeries, which increase the costs of patient study and management. We therefore emphasize on the need for additional studies with larger samples to establish diagnostic and therapeutic criteria that allow for the systematizing of patient care and treatment. The aim is to improve the quality of life of affected individuals, considering that the incidence of eosinophilic gastroenteritis has increased over the last decade [11].
